# Supplementary material for: A comprehensive in vivo screen of yeast farnesyltransferase activity reveals broad reactivity across a majority of CXXX sequences
Source: G3 (Bethesda). 2023 Apr 29;13(7):jkad094. doi: 10.1093/g3journal/jkad094 (PMC10320760; doi:10.1093/g3journal/jkad094)
Supplement: jkad094_Supplementary_Data [file jkad094_supplementary_data.zip › Supplemental_Material_Legends_G3-2023-404101.docx]

**Supplemental figure legends**

**Figure S1.** *Experimental scheme for genetic screening of Ydj1-CXXX variants assessed via next-generation sequencing (NGS).* A plasmid library encoding all 8000 Ydj1-CXXX variants was generated via ligation-based cloning method using PCR fragments derived using Trimer20 oligonucleotides (Integrated DNA Technologies, Newark, NJ). The library DNA was purified from *E. coli*, and plasmids were transformed into the *ydj1Δ* yeast strain. This strain cannot grow at temperatures >37 °C unless complemented by farnesylated Ydj1-CXXX variant. Populations of *ydj1∆/YDJ1-CXXX* transformants were incubated at permissive (25 °C) and selective (37 °C) temperatures, and plasmids recovered were subject to NGS analysis. Schematic illustration created with Microsoft PowerPoint and BioRender.com.

**Figure S2.** *Frequencies of CXXX sequences in naïve yeast library versus E. coli plasmid library.* CXXX frequencies from the naïve yeast and *E. coli* plasmid libraries were plotted against each other. No significant change in the frequency profile of individual sequences was observed between the two libraries. Libraries were not perfectly balanced, however, across all CXXX sequences; the extremes exhibited a ~10x range in relative abundance.

**Figure S3.** *3D analysis of top 5% of sequences from Ydj1-based and Ras-based data sets.* The data described in **Figures 2E, F** was plotted in 3D from the perspective of each axis of the 4D plot such that Lysine (K) is the nearest amino acid. The size of the spot is the 3^rd^ dimension and represents relative abundance. The perspectives are along the a_1_ axis (**A**, **B**), the a_2_ axis (**C**, **D**), and X axis (**E**, **F**) for the Ydj1-based data (**A**, **C**, **E**) and the Ras-based data (**B**, **D**, **F**).

**Figure S4.** *Thermotolerance assay of control sequences.* **A**) Gel-shift assays were performed for Ydj1 (CASQ) and His8-Nap1 (CKQS) in the presence and absence of FTase activity. Total yeast extracts were analyzed by SDS-PAGE and immunoblotting. The strains used were BY4741 (+FTase) and yWS1632 (*ram1*∆; -FTase). **B**) Thermotolerance assays were performed with the indicated Ydj1-CXXX variants as described for **Figure 4**. Abbreviations: Seq = Sequence, EF = Enrichment Factor, % = percent prenylation, N/A = not applicable.

**Figure S5.** *Full decision tree model. S*equence motif features were generated by one-hot encoding each of the variable CaaX motif sites. Additional binary features were included that describe whether the variable residue was within a given set of residues. To define these rules, all possible residue combinations up to 5 amino acids were considered, yielding a total of 65,097 features. While building the decision tree classifier, entropy was used to evaluate the quality of potential splits. Trees were allowed a maximum depth of 3 in order to determine more generalizable rules and all nodes were required to have a minimum of 50 samples. Samples refer to the number of CXXX variants from the previous node that fall within the category. Value refers to the distribution of CXXX variants that fall within specified EF ranges (0-1, 1-2, 2-3, 3-4, >4).

**File S1.** *Frequency of CXXX sequences observed in naive libraries*

**File S2.** *Enrichment factor results of Ydj1-based NGS*

**File S3.** *CKQX hits identified on UniProt*

**File S4.** *Heatmap analysis*
