# Supplementary figures and images for: A comprehensive in vivo screen of yeast farnesyltransferase activity reveals broad reactivity across a majority of CXXX sequences
Source: G3 (Bethesda). 2023 Apr 29;13(7):jkad094. doi: 10.1093/g3journal/jkad094 (PMC10320760; doi:10.1093/g3journal/jkad094)

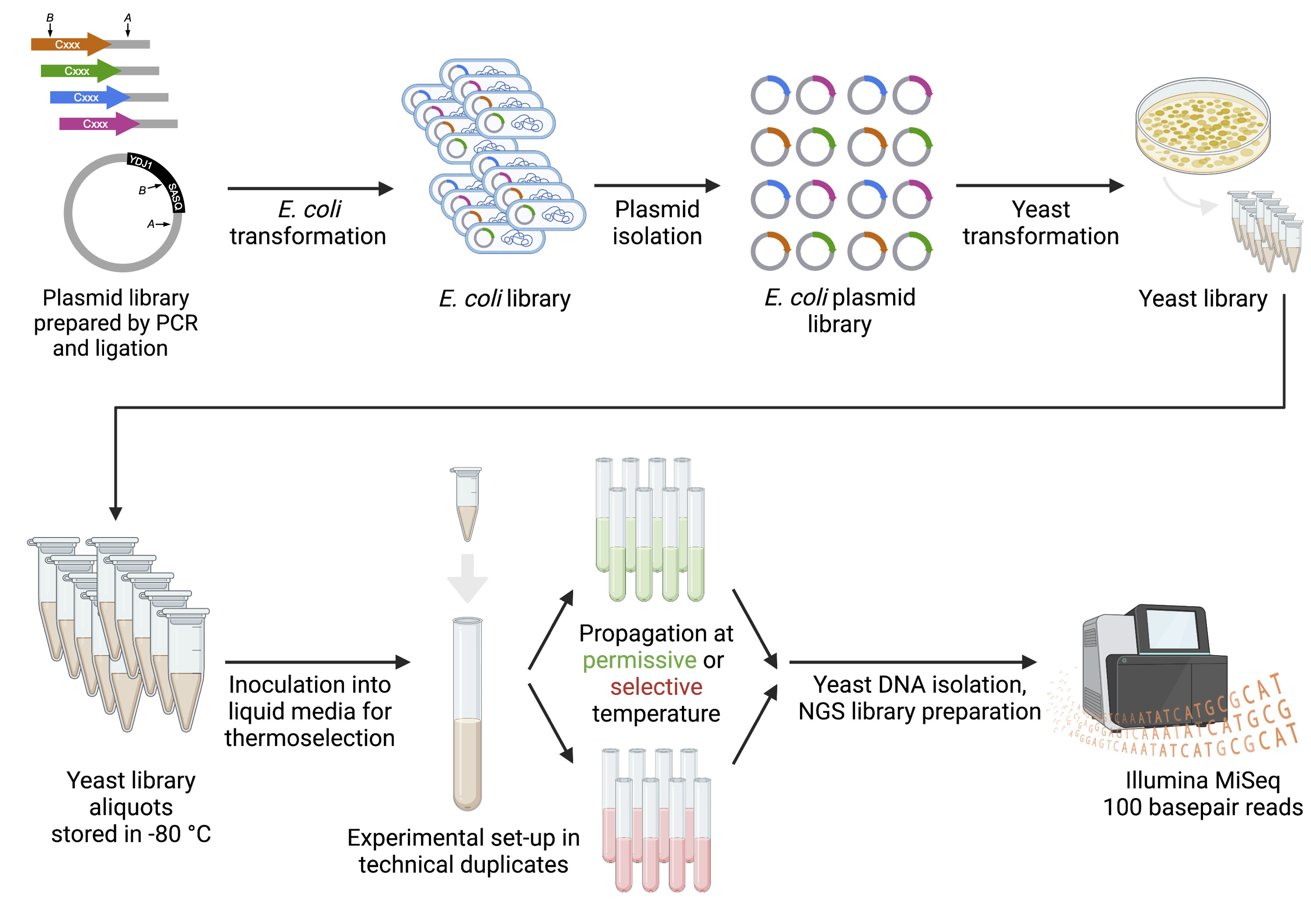

Supplement: jkad094_Supplementary_Data [file jkad094_supplementary_data.zip › Figure_S1_G3-2023-404101.tif]

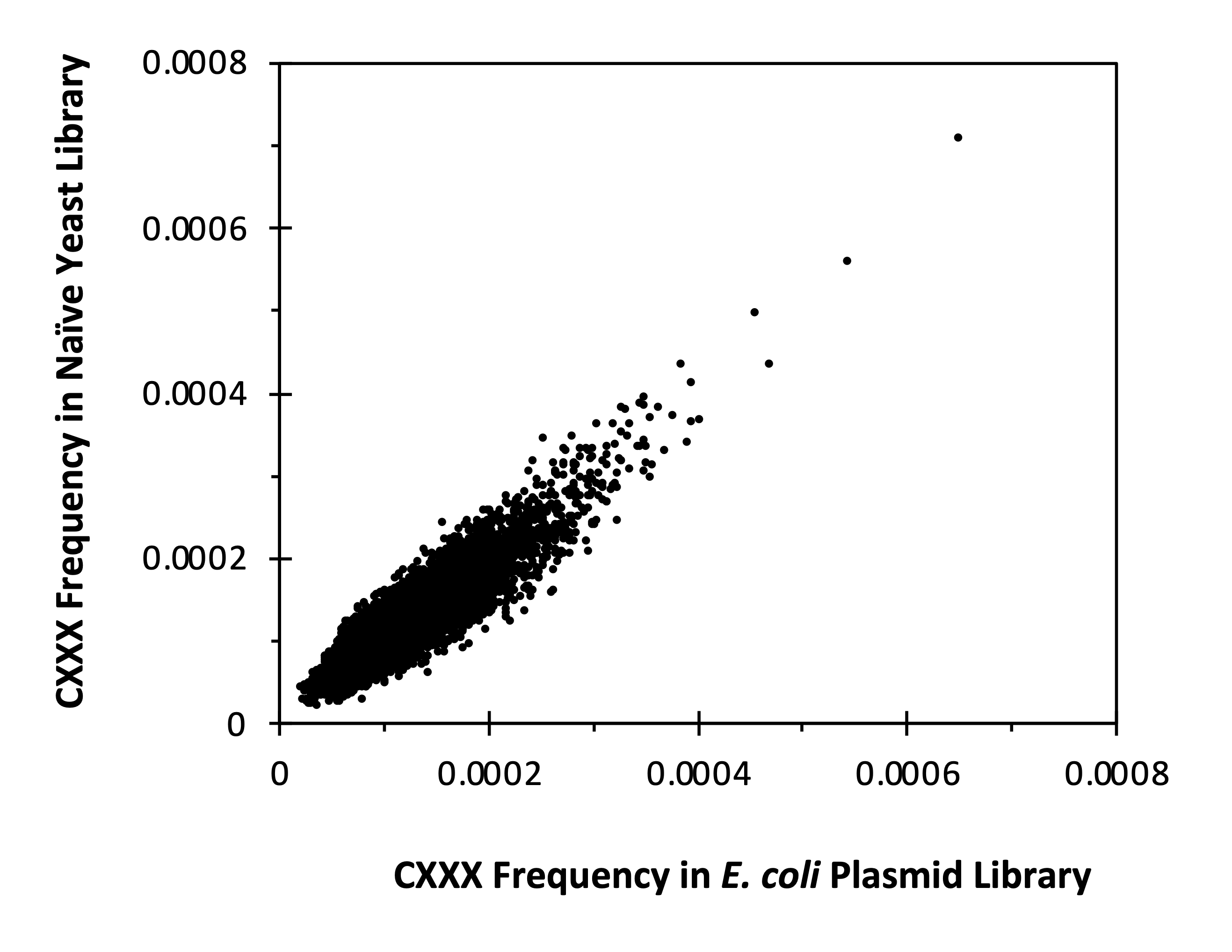

Supplement: jkad094_Supplementary_Data [file jkad094_supplementary_data.zip › Figure_S2_G3-2023-404101.tif]

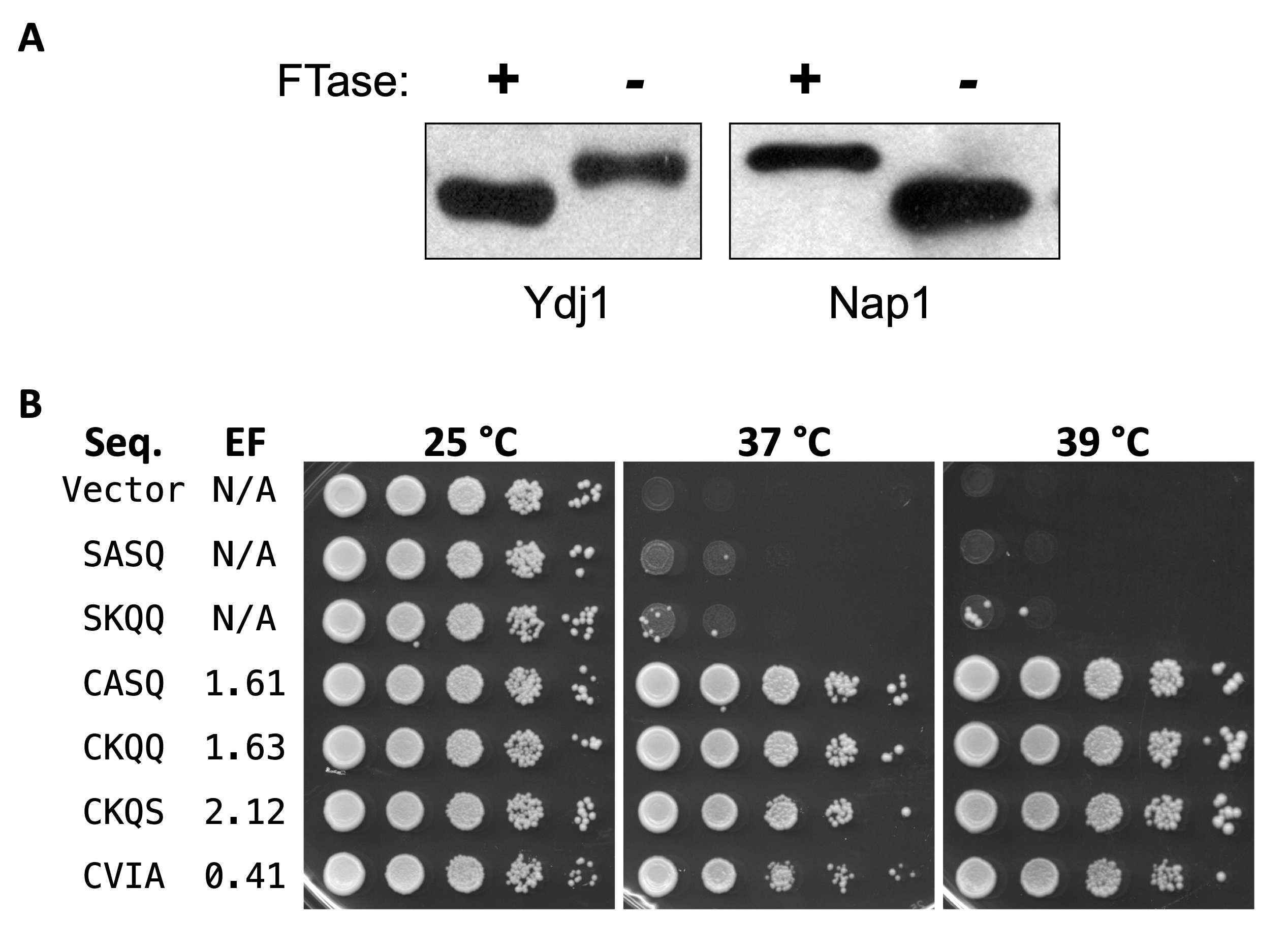

Supplement: jkad094_Supplementary_Data [file jkad094_supplementary_data.zip › Figure_S4_G3-2023-404101.tif]

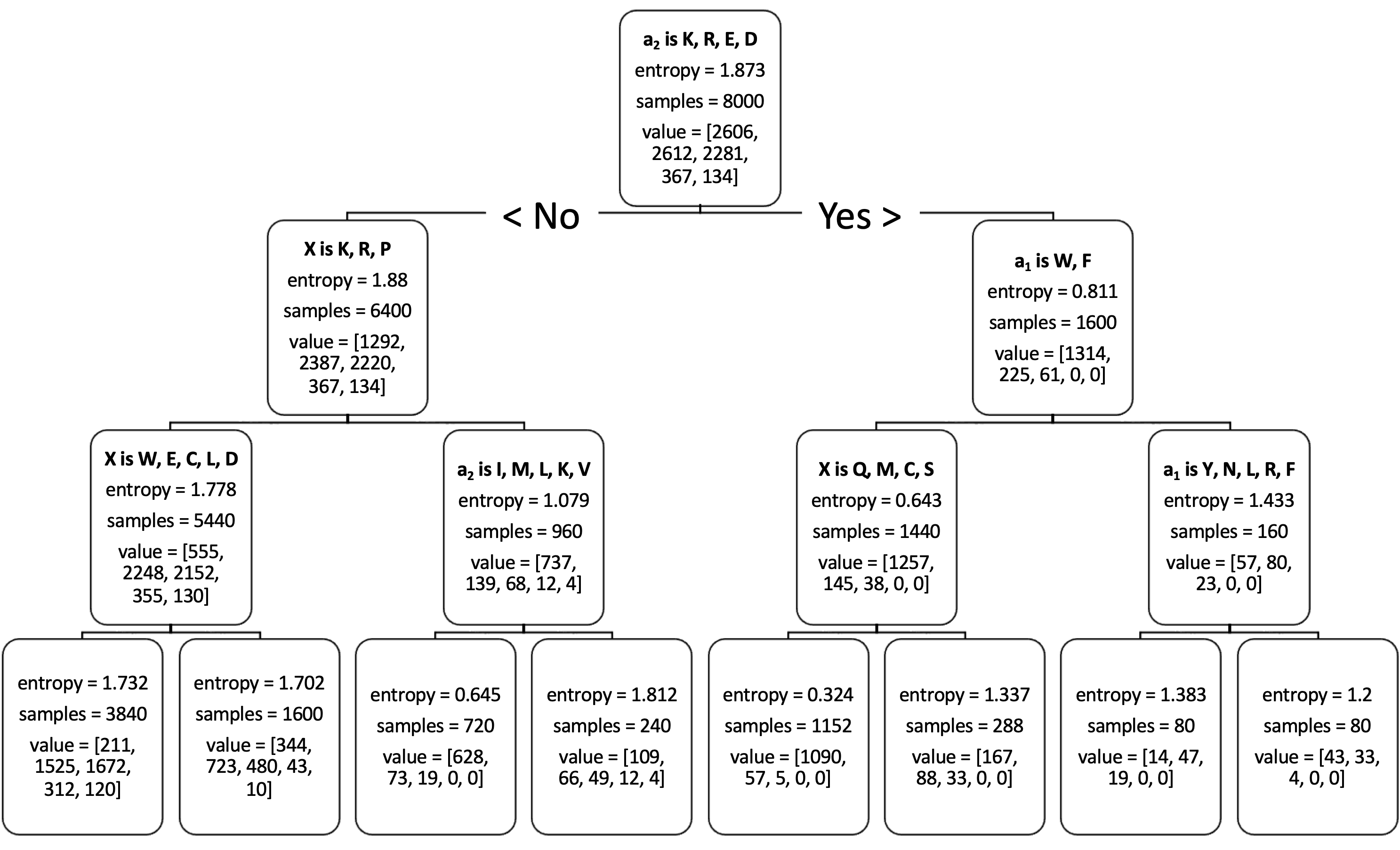

Supplement: jkad094_Supplementary_Data [file jkad094_supplementary_data.zip › Figure_S5_G3-2023-404101.tif]

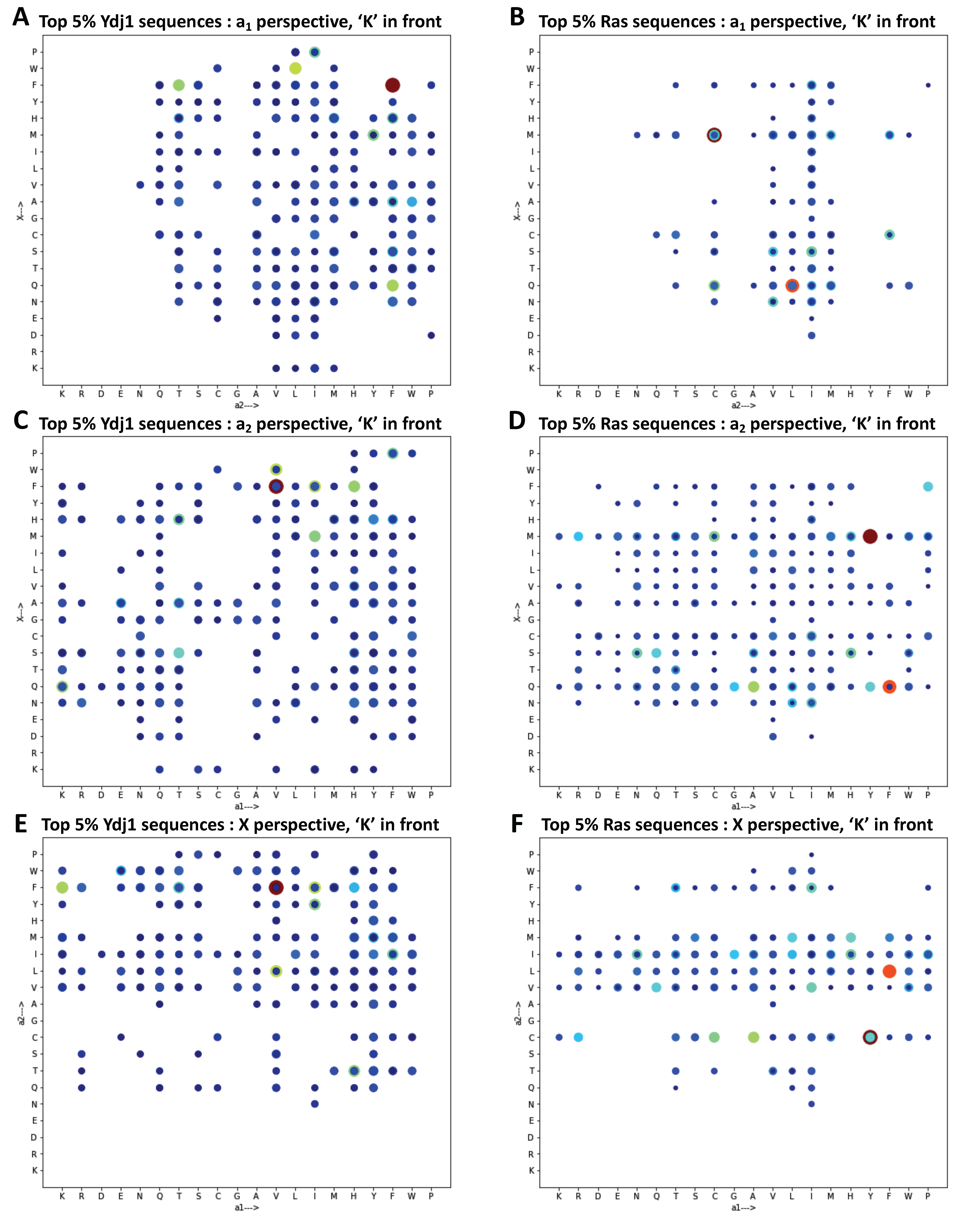

Supplement: jkad094_Supplementary_Data [file jkad094_supplementary_data.zip › Kim et al_FigureS3_ProofRevision_05042023.tiff]
